# Efficient engulfment of necroptotic and pyroptotic cells by nonprofessional and professional phagocytes

**DOI:** 10.1038/s41421-019-0108-8

**Published:** 2019-08-06

**Authors:** Jiongcong Lu, Wenke Shi, Bo Liang, Chang’an Chen, Rui Wu, Huaipeng Lin, Yingying Zhang, Jiahuai Han

**Affiliations:** State Key Laboratory of Cellular Stress Biology, Innovation Center for Cell Signaling Network, School of Life Sciences and School of Medicine, Xiamen University, Xiamen, 361005 Fujian China

**Keywords:** Apoptosis, Necroptosis

Dear Editor,

Engulfment of dead cells is essential for tissue homeostasis and prevention of inflammation. Classical theory is that this process is performed largely by professional phagocytes such as macrophages and dendritic cells (DC)^1^. However, increasing data suggest a role of neighboring nonprofessional cells in this process^2^. Uptake of apoptotic cells has been studied intensively, which involves recognition of apoptotic cells by receptors on engulfing cells that recognize the so-called ‘eat-me’ signals on the surface of the apoptotic cells^1,2^. Clearance of necrotic cells is thought of high importance to tissue homeostasis as necrotic cells could leak intracellular materials that might evoke inflammation. There are studies of necrotic cell clearance, but it is limited especially for the recently uncovered programmed necrosis including necroptosis and pyroptosis. The ‘eat-me’ signal phosphatidylserine (PS) can be detected on necroptotic cells^3,4^ and was believed to drive recognition and phagocytosis by professional phagocytes^1,5^. However, no direct evidence has been found to support that PS is required for the phagocytosis of necroptotic cells. Because of the interconversion between apoptosis and necroptosis or pyroptosis, mixed types of cell death often occurred in experiments, which could lead to misinterpretation of the results. In order to specifically induce a certain type of cell death, we used a dimerization/oligomerization system, which is based on antiestrogen 4-hydroxytamxifen (4-OHT)-induced homo-dimerization of hormone-binding domain’s G521R mutant (HBD*) of estrogen receptor, to artificially induce oligomerization of signaling molecules in cell death pathways in our previous studies^6,7^. Inducible oligomerization of caspase-8, mixed lineage kinase domain-like N-terminal domain (MLKL-ND), and gasdermin D N-terminal domain (GSDMD-N) in given cells such as NIH3T3 was used here to specifically induce apoptosis, necroptosis, and pyroptosis, respectively (Supplementary Fig. S1a). We detected PS exposure in all three types of cell death (Supplementary Fig. S1b). The PS positive cells were committed to death since they could not regrow (Supplementary Fig. S1c). We analyzed their phagocytosis by their live peers (nonprofessional phagocytes) using NIH3T3 cells and used heat-killed NIH3T3 cells as controls. Whereas heat-killed cells were engulfed very inefficiently, we observed the uptake of not only apoptotic cells but also necroptotic and pyroptotic cells (Fig. 1a, b; Supplementary Fig. S2a, c). In contrast to the common assumption that apoptosis would undergo the process of phagocytosis easily, necroptotic cells and pyroptotic cells were engulfed much more efficiently than apoptotic cells by their live peers (Fig. 1b; Supplementary Fig. S2a). The engulfment of necroptotic and pyroptotic cells can take place among the same type of cells and also between different types of cells (Supplementary Fig. S3a, b). More phagocytosis of TNF-induced necroptotic cells was also observed than that of apoptotic cells (Supplementary Fig. S3c). We then compared phagocytosis of necrotic cells with that of apoptotic cells by bone marrow-derived macrophages (BMDM), peritoneal macrophages, and bone marrow-derived dendritic cells (BMDC), and found that necroptotic and pyroptotic cells were engulfed more efficiently than apoptotic cells (Fig. 1c, d; Supplementary Figs. S2b, d and S3d–i). BMDM is the most potent phagocyte, and it also engulfs heat-killed cells with an efficiency similar to its uptake of apoptotic cells. The nonprofessional phagocytes showed similar phagocytic activity to that of BMDC, and we further analyzed engulfment of necroptotic cells by their live peers in more detail.

**Fig. 1:**
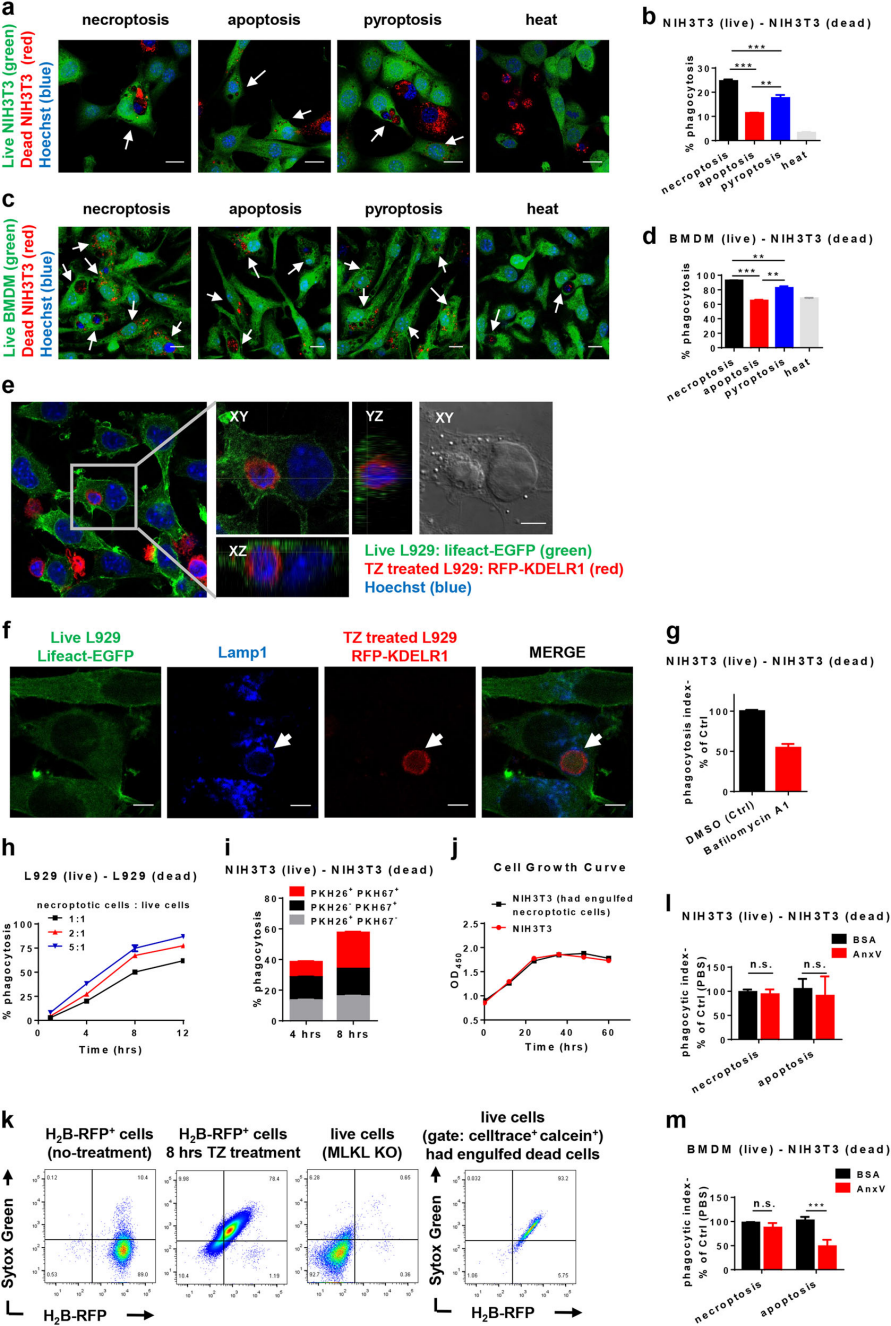
Engulfment of necroptotic and pyroptotic cells by nonprofessional and professional phagocytes. **a** NIH3T3 cells expressing MLKL-ND-HBD*, HBD*-Caspase-8, or GSDMD-N-HBD* were treated with 4-OHT (20 μM) for 10 min, 3 h and 10 min, respectively, to induce necroptosis, apoptosis, and pyroptosis. 4-OHT was then washed out and the cells were used as dying cells. PKH26 (cell membrane-labeling dye)-labeled dying cells (red) were co-cultured with CFSE (intracellular cell-tracing dye)-labeled live NIH3T3 cells (green) in the ratio of 1:1 for 4 h. Heat-killed NIH3T3 cells were generated by 65 °C incubation for 20 min and included in the experiment as accidental necrotic cell death control. Representative images are shown. Arrows indicate live cells that had engulfed dead cell(s). Scale bars, 20 μm. **b** Cells were treated as in **a** and analyzed using flow cytometry to measure the percentage of live CFSE^+^ NIH3T3 cells that had engulfed PKH26^+^ cells. **c** PKH26-labeled necroptotic, apoptotic, pyroptotic, and heat-killed cells as described in **a** were co-cultured with CFSE-labeled live BMDM in the ratio of 1:1 for 4 h. Representative images are shown. Arrows indicate live cells that had engulfed dead cell(s). Scale bars, 10 μm. **d** Phagocytosis of necroptotic, apoptotic, pyroptotic, and heat-killed NIH3T3 cells by BMDM was analyzed using flow cytometry by measuring the percentage of CD11b^+^ BMDM that had engulfed PKH26^+^ cells. **e**, **f** Lifeact-EGFP-expressing MLKL KO (necroptosis-resistant) L929 cells were co-cultured with RFP-KDELR1-expressing L929 cells in the presence of TZ (TNF and the caspase inhibitor z-VAD) for 6 h, and then counterstained with Hoechst (**e**) or immunostained for lysosome marker lamp1 (**f**). Arrows indicate the colocalization of the engulfed cell with lamp1. Scale bars, 5 μm. **g** NIH3T3 cells (live) were pretreated with DMSO or Bafilomycin A1 for 30 min before co-culture with necroptotic NIH3T3 cells as described in **a** in the ratio of 1:1. The phagocytosis was measured by flow cytometry. **h** CFSE-labeled MLKL KO L929 cells (live) were co-cultured with TZ-treated PKH26-labeled L929 cells (dead) in different ratios and the phagocytosis was analyzed by flow cytometry at indicated time points. **i** Celltrace violet-labeled NIH3T3 cells (live) were co-cultured with PKH26- and/or PKH67-labeled necroptotic NIH3T3 cells (dead) as described in **a** in the ratio of 1:1. PKH26^+^ and/or PKH67^+^ cells in live NIH3T3 (celltrace^+^) were analyzed by flow cytometry at indicated time points. **j** PKH67-labeled NIH3T3 cells (live) were co-cultured with PKH26-labeled necroptotic cells as described in **a** for 4 h. Then PKH67^+^ PKH26^−^ NIH3T3 cells and PKH67^+^PKH26^+^ NIH3T3 cells were sorted by FACS (fluorescence-activated cell sorting) and the growth in the next 60 h was measured using a cell counting kit 8 (MCE). **k** Celltrace far red-labeled MLKL KO L929 cells (live) were co-cultured with H_2_B-RFP-expressing L929 cells with or without TZ in the presence of Sytox Green for 8 h. The cells were then stained with live cell dye calcein blue and the live cells having engulfed dead cell(s) were gated by triple positive of calcein, celltrace, and RFP using flow cytometry (right panel). Left three panels show the live and dead H_2_B-RFP-expressing L929 cells as well as live MLKL KO cells for reference. **l**, **m** Necroptotic or apoptotic NIH3T3 cells treated as in **a** were incubated with BSA or Annexin V (AnxV, 100 μg/mL) for 30 min before co-culture with NIH3T3 (**l**) or BMDM (**m**) for 4 h in the ratio of 1:1 (The final concentration of Annexin V and BSA was 50 μg/mL). The phagocytosis was measured by flow cytometry. Data represent mean ± SD. ***P < 0.001; **P < 0.01; *P < 0.05; n.s., not significant. Student’s *t* test

In Fig. 1e, a live lifeact-EGFP (enhanced green fluorescent protein)-expressing L929 cell is shown, which has engulfed an intact necroptotic RFP (red fluorescent protein)-KDELR1-expressing L929 cell. Lifeact is an actin-binding 17-amino-acid peptide that does not interfere with actin dynamics^8^, and KDELR1 is an endoplasmic reticulum-localized protein^9^. Necroptosis of RFP L929 cells was induced by TNF in the presence of pan caspase inhibitor zVAD (TZ treatment). The engulfment process is shown in Supplementary Movies S1 and S2. Like classical phagocytosis, pseudopods form on live cells (green), probing for necroptotic cells (red) in the environment. Once a necroptotic cell is touched by a pseudopod, it is pulled towards the live cell and an engulfment begins as a phagocytic cup-like structure gradually wrapping around the dead cell (Supplementary Fig. S4 and Supplementary Movie S1). Interestingly, live cells not always succeed in engulfing the intact necroptotic prey, as we observed that two live cells both began to engulf a single necroptotic cell and ripped the dead cell apart (Supplementary Movie S2). The engulfed cell went to lysosome as indicated by its colocalization with Lamp1 (Fig. 1f). Inhibition of vacuolar H^+^-ATPase by balifomycin A1 impaired the phagocytosis (Fig. 1g).

We incubated necroptotic L929 cells with live L929 cells in different ratios and measured the uptake of dead cells at different time points (Fig. 1h). The uptake of dead cells was time-dependently increased and higher ratio of necroptotic cells also increased uptake. The ratio of live cells that has taken necroptotic cell(s) reached almost 100%, indicating that every live cell is capable to engulf necroptotic cells. Extending co-culture time also increased the phagocytosis of apoptotic cells (Supplementary Fig. S3j). To address whether one live cell could engulf more than one necroptotic cell, we used PKH67 or PKH26 to stain necroptotic NIH3T3 cells separately and then incubated their mixture with live cells. We detected certain amount of PKH67 and PKH26 double positive live cells, indicating that an individual live cell can engulf more than one necroptotic cell (Fig. 1i). Although the engulfed dead cell could provide nutrient for the live peers, we did not observe any difference in proliferation between cells that had taken or had not taken dead cells (Fig. 1j). We next determined whether the engulfment of necroptotic cells by their live peers occurred before or after their plasma membrane disruption. H_2_B is a nuclear protein that stays in cells even when the plasma membrane is broken. We used necroptosis-resistant MLKL knockout (KO) L929 cells as live cells and H_2_B-RFP (fusion protein of histone 2 B and red fluorescent protein)-expressing L929 cells to generate necroptotic cells. Celltrace-labeled MLKL KO L929 cells and H_2_B-RFP-expressing L929 cells were incubated individually or together in the presence or absence of TZ and cell-impermeant nucleic acid dye Sytox Green. Cells were collected and stained with another live cell dye calcein blue, and then analyzed by flow cytometry. The live cells that had engulfed dead cell(s) were gated using RFP positive plus double positive of celltrace and calcein blue. Sytox Green and RFP intensities of gated cells were shown in Fig. 1k (right panel). Almost all the live cells that had engulfed RFP-positive cell were Sytox Green positive, indicating the loss of plasma membrane integrity occurred before dead cells being engulfed.

It is well known that blocking PS by Annexin V can efficiently inhibit phagocytosis of apoptotic cells^10^. We found that while Annexin V blocked engulfment of apoptotic cells by BMDM or BMDC, it had no effect on the uptake of necroptotic and pyroptotic cells by its peers (Fig. 1l, m; Supplementary Fig. S5a–c). We then collected supernatants of necroptotic and pyroptotic cells and found that the supernatants had no effect on the phagocytosis of apoptotic cells, excluding the possibility that necrotic cells release factor(s) that blocks classic phagocytosis. We also knocked down calreticulin (CRT), another crucial ‘eat-me’ signal in apoptosis^1,2^, and found it did not affect the engulfment of necroptotic cells by BMDC and NIH3T3 cells (Supplementary Fig. S5d, e). The addition of soluble CRT peptide that competitively bind CRT receptor(s) also showed no blocking effect (Supplementary Fig. S5f). Expression of a PS receptor Tim4^1^ in NIH3T3 cells enhanced their ability to engulf both necroptotic and apoptotic NIH3T3 cells but Annexin V had no inhibitory effects on the enhanced engulfment, suggesting the involvement of other ligand(s) of Tim4 (Supplementary Fig. S5g). We then performed a series of experiments using different inhibitors to compare the engulfment of necroptotic and pyroptotic NIH3T3 cells by NIH3T3 and BMDC. Inhibition of actin filaments by cytochalasin B suppressed the engulfment by both NIH3T3 and BMDC (Supplementary Fig. S6a), so did inhibition of PI3K by wortmanin (Supplementary Fig. S6b) and inhibition of Rac 1 by EHop-016 (Supplementary Fig. S6c). Inhibition of integrin by SB273005 and inhibition of ROCK by Y27632 had no effect on the phagocytosis by both types of cells (Supplementary Fig. S6d, e). In addition, engulfment of necroptotic and pyroptotic NIH3T3 cells by NIH3T3 but not by BMDC is heavily dependent on Ca^2+^ (Supplementary Fig. S6f–i). Thus, engulfment of necroptotic and pyroptotic cells should be mediated by other ligand-receptor(s) and there are also differences in the mechanisms used by professional and nonprofessional phagocytes.

It was unexpected that the phagocytosis of necroptotic and pyroptotic cells is more efficient than that of apoptotic cells. Since necrosis is inflammatory, high efficiency of the engulfment of necroptotic and pyroptotic cells could be a mechanism to prevent inflammation. It is interesting to find out the high efficiency of phagocytosis of necroptotic and pyroptotic cells by nonprofessional phagocytes. Nonprofessional phagocyte-mediated engulfment of apoptotic cells is involved in the clearance of apoptotic cells during foot plate development^11^, and of airway apoptotic epithelial cells under allergen stimulation^12^. The phagocytosis of necrotic cells by nonprofessional phagocyte is highly likely to occur in vivo. If so, the engulfment of necrotic cells by their neighborhood nonprofessional phagocytes should be the very first line of defense against necrotic cell-induced local inflammation.

## Supplementary information

Supplementary Information

Supplementary Movie S1

Supplementary Movie S2

## Supplementary information

**Supplementary Information** accompanies the paper at (10.1038/s41421-019-0108-8).
