# Supplementary material for: Efficient engulfment of necroptotic and pyroptotic cells by nonprofessional and professional phagocytes
Source: Cell Discov. 2019 Aug 6;5:39. doi: 10.1038/s41421-019-0108-8 (PMC6796833; doi:10.1038/s41421-019-0108-8)
Supplement: Supplementary file 1 — Supplementary Information [file 41421_2019_108_MOESM1_ESM.pdf]

Materials and Methods

Cell culture

To obtain bone marrow derived macrophage (BMDM), bone marrow collected from mouse (C57BL/6J, 8–10 weeks old) femurs and tibias was plated on sterile petri dishes and incubated for 7 days in Dulbecco's modified Eagle's medium (DMEM) (Gibco) containing 10% (vol/vol) heat-inactivated FBS, 100 units/ml, penicillin, 100 mg/ml streptomycin and 30% (vol/vol) conditioned medium from mouse L929 fibroblasts.

Peritoneal macrophages were collected from mice 4 or 5 days after thioglycollate (sigma, T9032) injection i.p.. Then peritoneal macrophages were cultured in endotoxin-free RPMI1640 (Gibco) with 10% (vol/vol) fetal bovine serum for use.

To obtain bone marrow derived dentric cell (BMDC), bone marrow collected from mouse (C57BL/6J, 8–10 weeks old) femurs and tibias was plated on sterile petri dishes and incubated for 7 days in RPMI1640 containing 10% (vol/vol) heat-inactivated FBS, 100 units/ml penicillin, 100 mg/ml streptomycin, $5x10^{-5}$ M β-Me and 15% (vol/vol) conditioned medium from GM-CSF-expressing mouse X-63 B cell.

L929 cell, NIH3T3 cell, MCA205 cell and MDCK cell were cultured in DMEM containing 10% (vol/vol) FBS.

Reagents and antibodies

Mouse TNF alpha (34-8321-85) and Annexin V Apoptosis Detection Kit APC (88-8007-72) were from eBioscience. Z-VAD-FMK (627610) was from Calbiochem. Smac mimetic (SM-164) was from APExBIO. GSK872 (5.30389.0001) was from Millipore. CellTrace CFSE Cell Proliferation Kit (C34554), CellTrace Far Red Cell Proliferation Kit (C34572), CellTrace Violet Cell Proliferation Kit (C34557), SYTOX Green Nucleic Acid Stain (S7020), Alexa Fluor 647 phalloidin (A22287) and Calcein Blue (C1429) were from Invitrogen. 4 hydroxytamoxifen (4-OHT) (H7904), PKH67 Green Fluorescent Cell Linker Kit (MINI67), PKH26 Red Fluorescent Cell Linker Kit (MINI26) and BSA (A3311) were from Sigma. Cell counting kit 8 (hy-k0301) was from MCE. Bafilomycin A1 (1334) was from Tocris. Cytochalasin B (ab143482) was from Abcam. Wortmannin (S2758), Ehop-016 (S7319), SB273005 (S7540) and Y-27632 2HCl (S1049) were from Selleckchem. Purified Recombinant Annexin V (556416) was from BD Pharmingen. Calreticulin Blocking Peptide (3077BP) was from biovision. Anti-Lamp1 (ab13523) and anti-Calreticulin (ab92516) were from Abcam. FITC anti-mouse/human CD11b (101205) and Brilliant Violet 421 anti-mouse CD11c (117329) were from Biolegend.

Phagocytosis assay

Cultured cells were labeled with indicated dye as mentioned in the main text. Live NIH3T3, L929, macrophages and dendritic cells were seeded in 48-well plate at a density of 1.5 x 10^5 per well, and 1.5 x 10^5 of indicated dying/dead cells were added to co-incubation for indicated time. Then cells were detached using EDTA or trypsin-EDTA, washed by PBS and collected to analyze. For professional phagocytes, cells were marked by anti-CD11b (macrophages) or anti-CD11c (dendritic cells) antibodies. Phagocytosis was measured by flow cytometry using Fortessa X-20 (BD biosciences). The percentage of live CellTrace$^+$ NIH3T3/L929 cells, CD11b$^+$ macrophages or CD11c$^+$ dendritic cells that had engulfed PKH$^+$ cells was calculated (PKH$^+$ CellTrace $^+$/ CellTrace$^+$, PKH$^+$ CD11c $^+$/ CD11c $^+$ or PKH$^+$ CD11b $^+$/ CD11b $^+$). In some experiments, cells were plated on coverslips or glass-bottom dishes for co-incubation and images were generated using microscopy.

Microscopy

For fixed cell imaging, cells were plated on coverslips pre-coated with poly-L-lysine. In some experiments, cells were stained first with CFSE or PKH26/67 as indicated. After treatment, cells were washed with PBS followed by fixation with 4% paraformaldehyde in PBS. In some experiments, cells were then permeabilized with 0.2% Triton X-100 in PBS, blocked with 3% BSA in PBS and stained with anti-Lamp1 antibody and secondary antibody, or stained with Alexa Fluor 647 phalloidin. Cells were counterstained with Hoechst to visualize nuclei. Images were generated using Zeiss LSM 780 confocal microscope or Delta-Vision OMX v4 (GE Healthcare).

For time-lapse microscopy live cell imaging, PKH26-labeled L929 cells and lifeact-EGFP-expressing MLKL KO L929 cells were seeded in 35mm glass-bottom dishes in the ratio of 1:1. TZ was added to medium to induce necroptosis. Then imaging was carried out using Zeiss LSM 780 confocal microscope.

RNA interference

Lentiviral-shRNAs were constructed into pLV-H1-EF1α-puro vector following the manufacturer's instruction (Biosettia). MLKL-ND-HBD*-expressing NIH3T3 cells were infected with lentiviruses carrying calreticulin (shCRT) shRNA or non-target shRNA (shSCR). The CRT shRNA target sequence was 5'GCAGACCCTGCCATCTATTTC-3'. Knockdown efficiency was measured by immunoblotting using anti- calreticulin antibody.

Supplementary Fig.S1

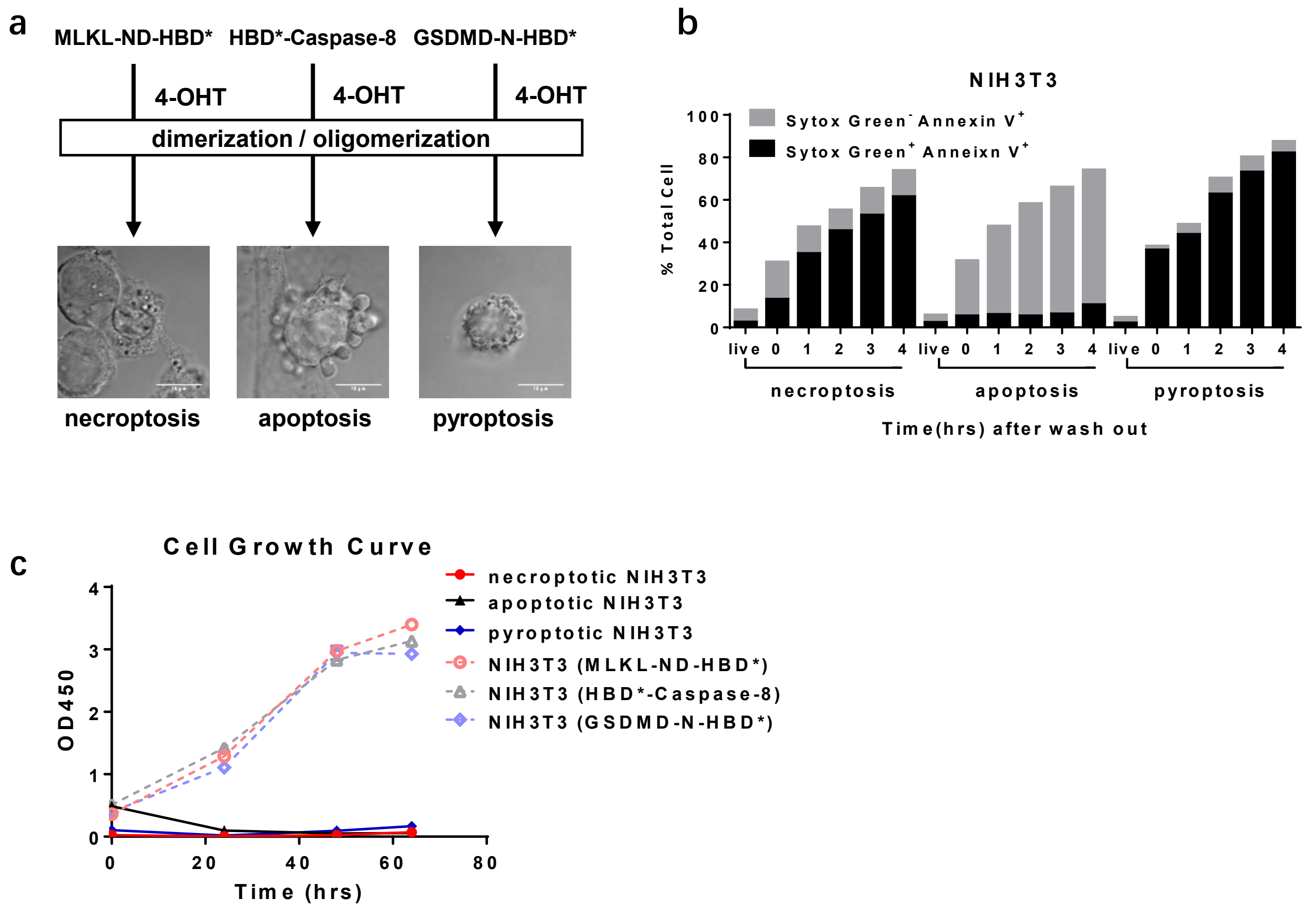

Supplementary Fig. S1 Specific induction of necroptosis, apoptosis or pyroptosis using dimerization system of HBD*.

(a) Schematic and morphology representation of HBD* dimer system induced necroptosis, apoptosis and pyroptosis. MLKL-ND, MLKL 1-190 aa. Caspase-8, caspase-8 198-480 aa. GSDMD-N, GSDMD 4-276 aa. Scale bars, 10μm.

(b) NIH3T3 cells expressing MLKL-ND-HBD*, HBD*-Caspase-8 or GSDMD-N-HBD* were induced to necroptosis, apoptosis and pyroptosis by 4-OHT treatment for 10 mins, 3 hrs and 10 mins, respectively. After treatment, 4-OHT was washed out and the cells were further incubated at 37°C for different periods of time. The cells were stained with Annexin V and Sytox Green then analyzed by flow cytometry.

(c) NIH3T3 cells expressing MLKL-ND-HBD*, HBD*-Caspase-8 or GSDMD-N-HBD* were left untreated or induced to necroptosis, apoptosis and pyroptosis by 4-OHT treatment for 10 mins, 3 hrs and 10 mins, respectively. After treatment, 4-OHT was washed out and the cells were further incubated at 37°C. The growth in the next 64 hrs was measured using cell counting kit 8 (MCE).

Supplementary Fig.S2

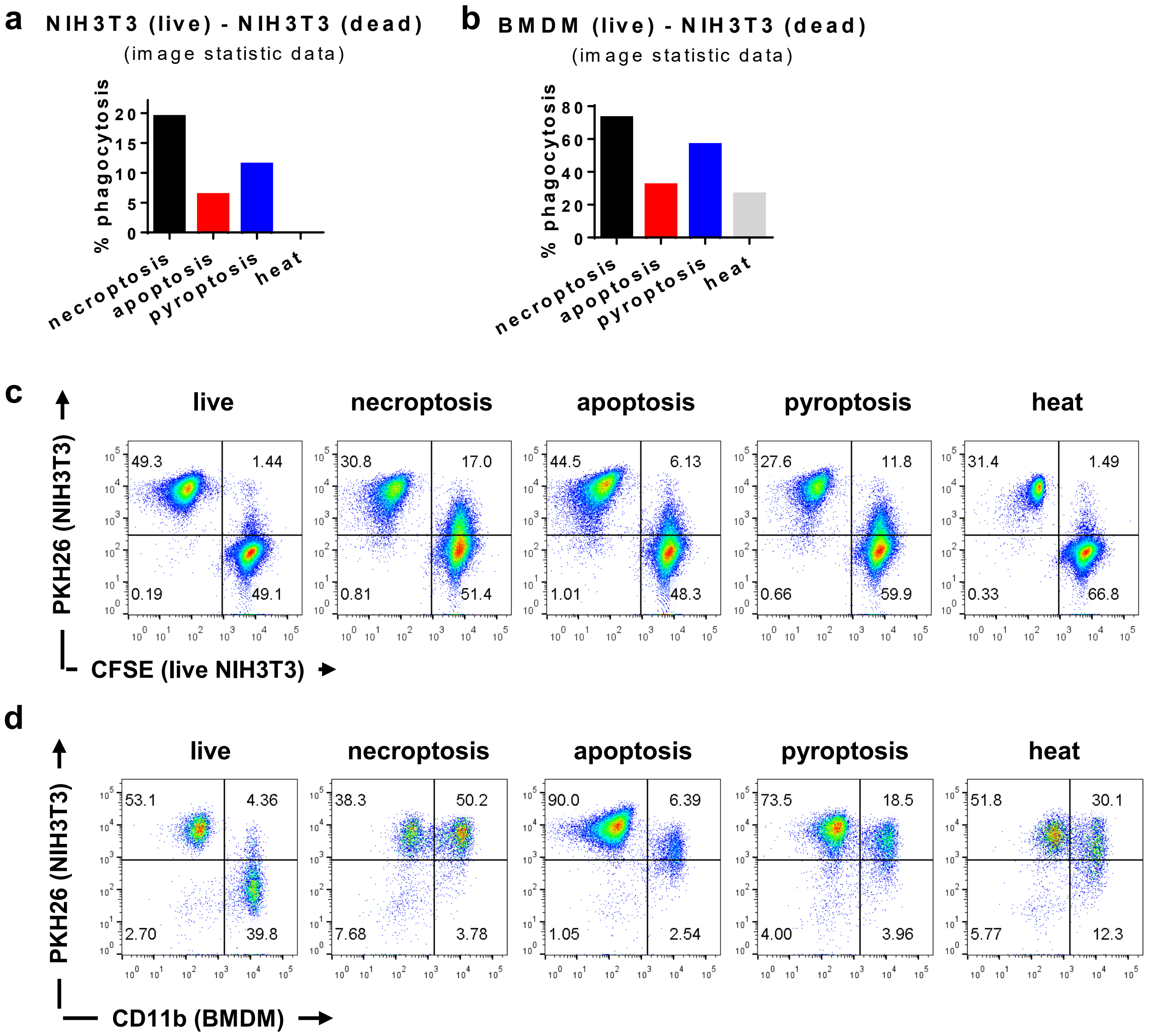

Supplementary Fig. S2 Quantitative analysis related to Figure 1a, 1c, 1b, and 1d

(a, b) Statistics analysis of source images data in **Fig. 1a (a)** and **Fig. 1c (b)**.

(c, d) Raw FACS plots data of **Fig. 1b (c)** and **Fig. 1d (d).**

Supplementary Fig.S3

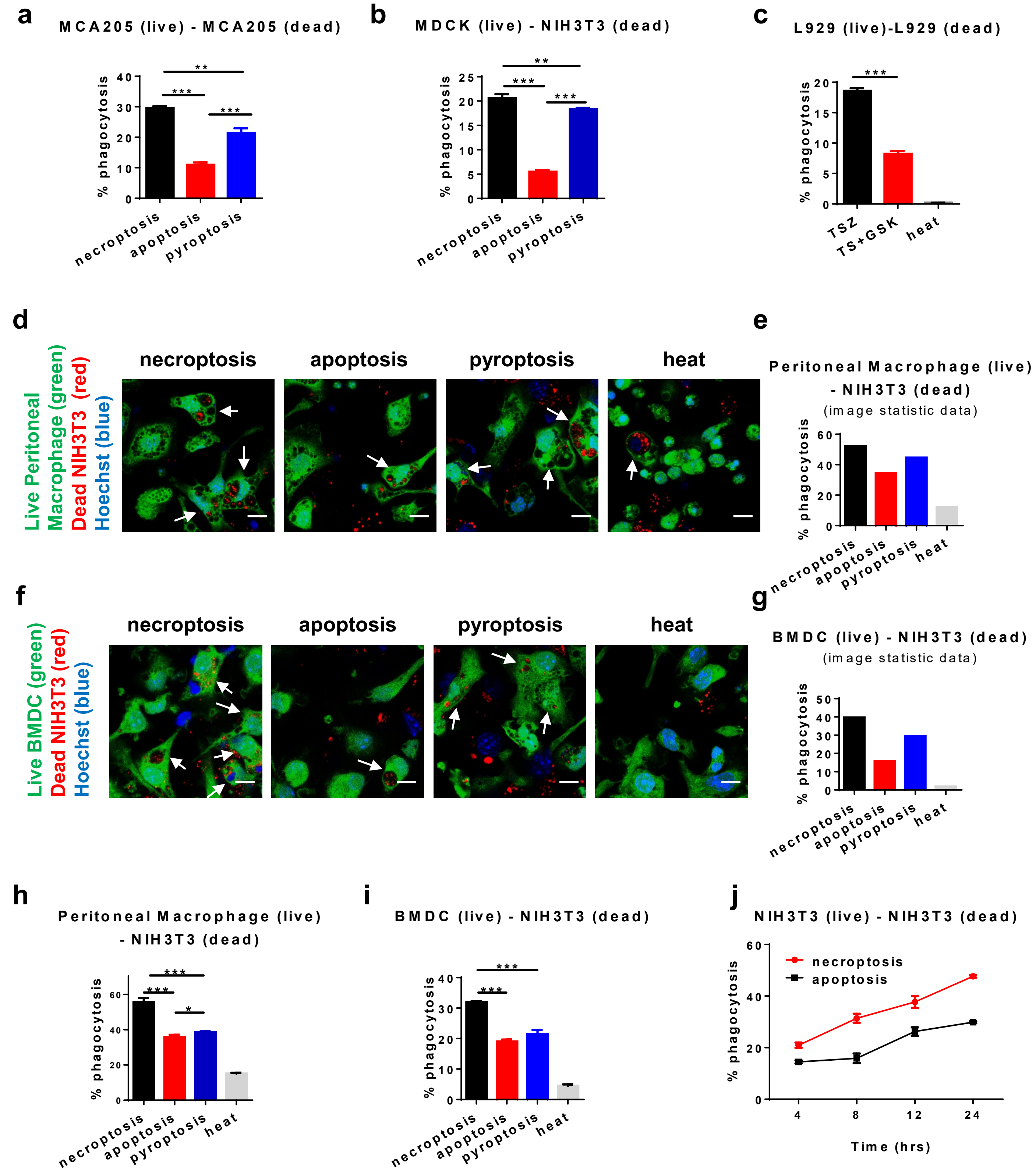

Supplementary Fig. S3 Engulfment of necroptotic, apoptotic and pyroptotic cells by different types of cells.

(a) CFSE-labeled MCA205 cells were co-cultured with PKH26-labeled necroptotic, apoptotic, or pyroptotic MCA205 cells in the ratio of 1:1 for 4 hrs. Necroptosis, apoptosis, or pyroptosis was induced by oligomerization inducer in Fv-Caspase-8, Fv-RIP3 or GSDMD-N-HBD* expressing cells. Phagocytosis was analyzed using flow cytometry.

(b) CFSE-labeled MDCK cells were co-cultured with necroptotic, apoptotic or pyroptotic NIH3T3 cells treated as in **Fig. 1a** in the ratio of 1:1 for 4 hrs. Phagocytosis was analyzed using flow cytometry.

(c) CFSE-labeled MLKL KO L929 cells were co-cultured with TNF+SMAC mimetic+zVAD induced necroptotic L929, TNF+SMAC mimetic+GSK872 induced apoptotic L929 or heat-killed L929 in the ratio of 1:1 for 4 hrs. Phagocytosis was analyzed using flow cytometry.

(d) PKH26-labeled necroptotic, apoptotic, pyroptotic and heat-killed cells treated as in **Fig. 1a** were co-cultured with CFSE-labeled peritoneal macrophage in the ratio of 1:1 for 4 hrs. Representative confocal microscopy images are shown. Arrows indicate live cells that had engulfed dead cell(s). Scale bars, 10μm.

(e) Statistical analysis of data of source images in **(d)**.

(f) PKH26-labeled necroptotic, apoptotic, pyroptotic and heat-killed cells treated as in **Fig. 1a** were co-cultured with CFSE-labeled BMDC in the ratio of 1:1 for 4 hrs. Representative confocal microscopy images are shown. Arrows indicate live cells that had engulfed dead cell(s). Scale bars, 10μm.

(g) Statistical analysis of data of source images in **(f)**.

(h) PKH26-labeled necroptotic, apoptotic, pyroptotic and heat-killed cells treated as in **Fig. 1a** were co-cultured with peritoneal macrophage in the ratio of 1:1 for 4 hrs. Phagocytosis was analyzed using flow cytometry by measuring the percentage of $CD11b^+$ peritoneal macrophages that had engulfed $PKH26^+$ NIH3T3 cells.

(i) PKH26-labeled necroptotic, apoptotic, pyroptotic and heat-killed cells treated as in **Fig. 1a** were co-cultured with BMDC in the ratio of 1:1 for 4 hrs. Phagocytosis was analyzed using flow cytometry to measure the percentage of $CD11c^+$ BMDC that had engulfed $PKH26^+$ cells.

(j) PKH26-labeled necroptotic and apoptotic cells treated as in **Fig. 1a** were co-cultured with CFSE-labeled live NIH3T3 in the ratio of 1:1 and phagocytosis was analyzed by flow cytometry at indicated time points.

Data represent mean ± SD. ***$P < 0.001$; **$P < 0.01$; *$P < 0.05$; ns, not significant. Student's t test.

Supplementary Fig. S4

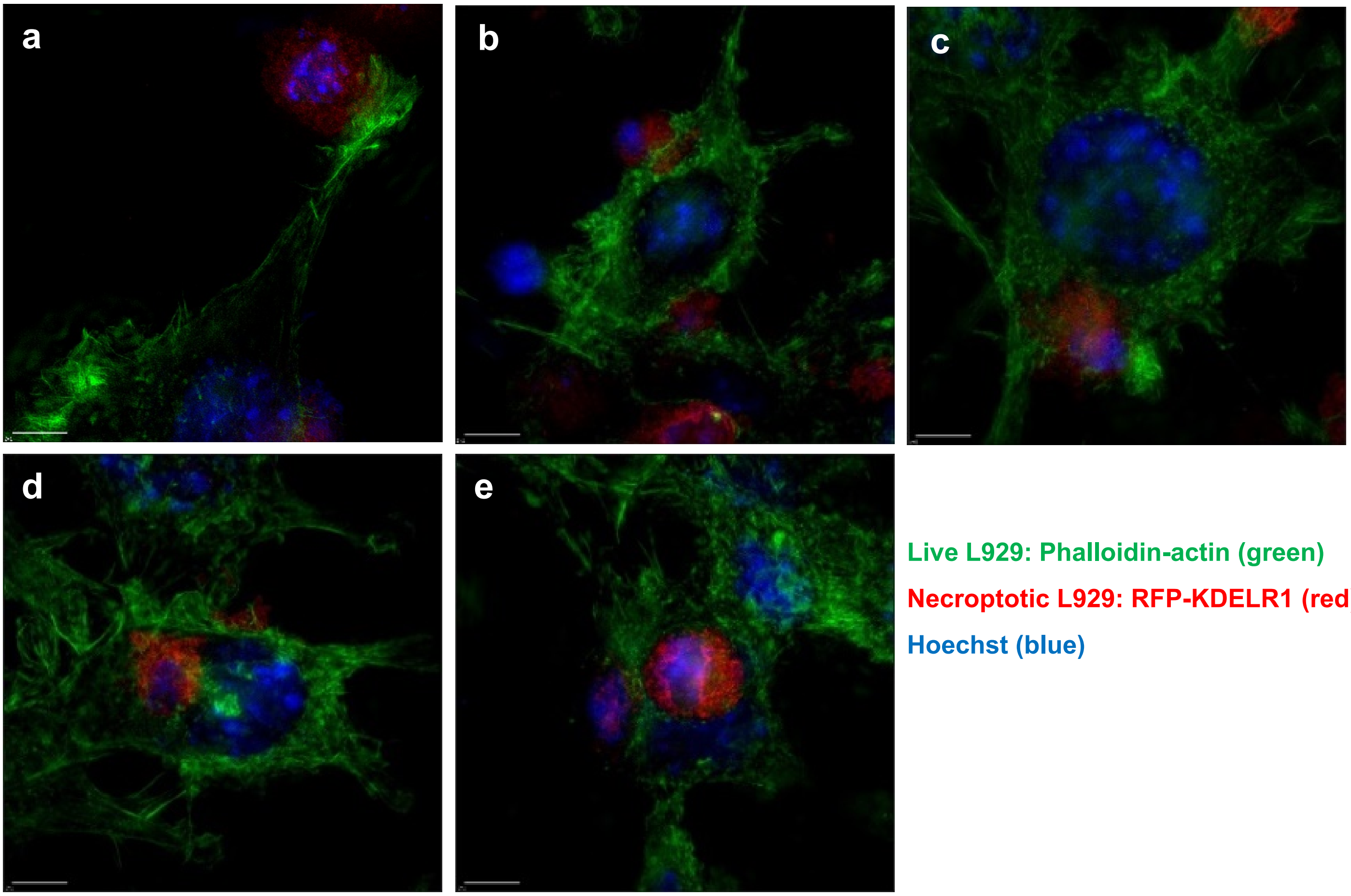

Live L929: Phalloidin-actin (green)

Necroptotic L929: RFP-KDELR1 (red)

Hoechst (blue)

Supplementary Fig. S4 Engulfment of necroptotic L929 by live L929.

Structured Illumination Microscopy (SIM) image of MLKL KO L929 cells co-cultured with RFP-KDELR1-expressing L929 cells in the presence of TZ for 6 hrs. Cells were stained with phalloidin and counterstained with Hoechst after treatment. Live cells catching **(a, b)**, engulfing **(c, e)** or having engulfed **(d, e)** necroptotic cells were shown. Scale bars, 5μm.

Supplementary Fig.S5

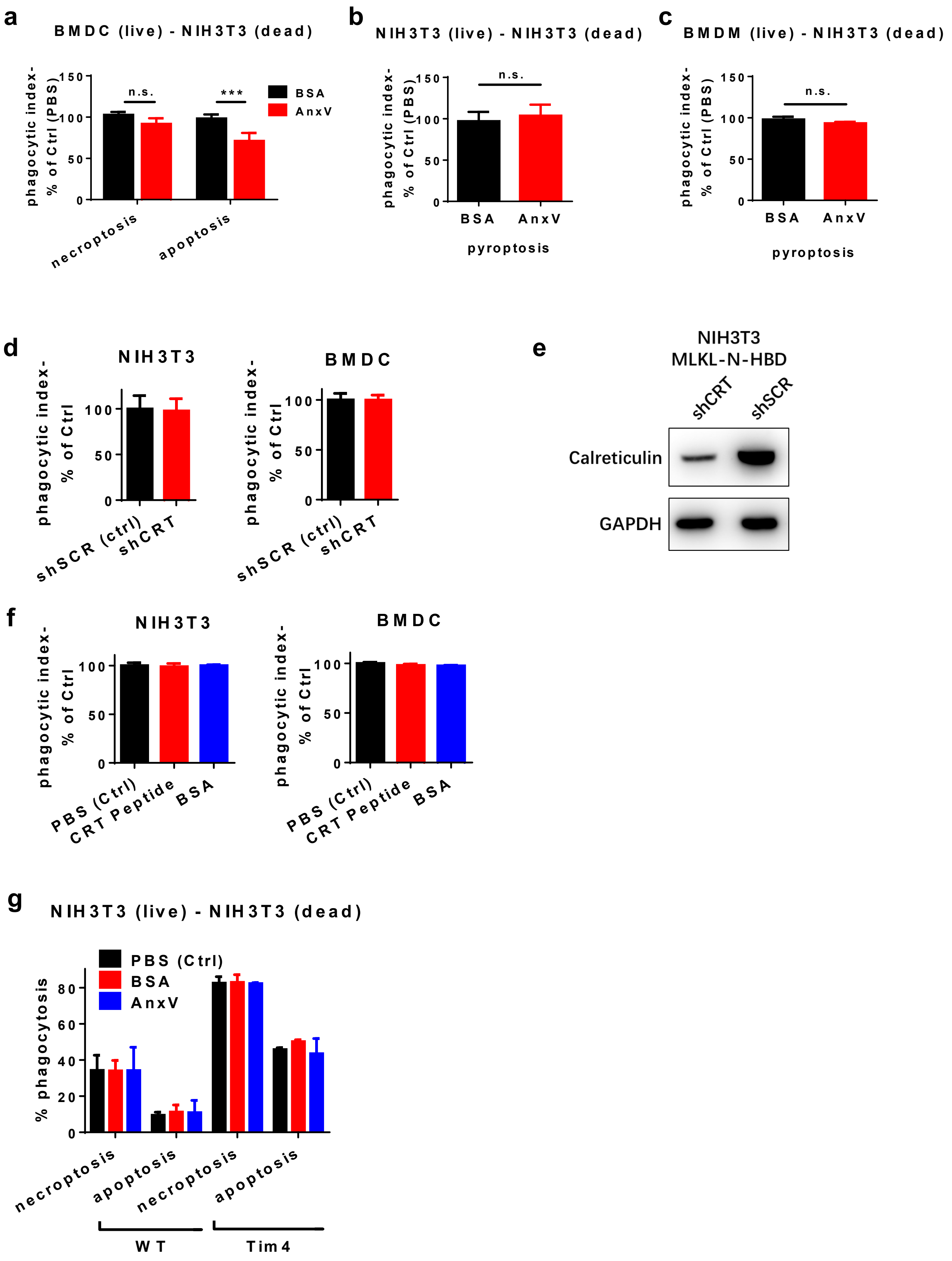

Supplementary Fig. S5 Canonical 'eat-me' signal phosphatidylserine and calreticulin are not involved in the engulfment of necroptotic cells.

(a) Necroptotic or apoptotic NIH3T3 cells treated as in **Fig. 1a** were pretreated with BSA or Annexin V (AnxV, 100μg/ml) for 30 mins before co-culture with BMDC for 4 hrs in the ratio of 1:1 (The final concentration of Annexin V and BSA was 50μg/ml). Then phagocytosis was analyzed by flow cytometry.

(b, c) Pyroptotic NIH3T3 cells treated as in **Fig. 1a** were pretreated with BSA or Annexin V (AnxV, 100μg/ml) for 30 mins before co-culture with NIH3T3 **(b)** or BMDM **(c)** for 4 hrs in the ratio of 1:1 (The final concentration of Annexin V and BSA was 50μg/ml). Then phagocytosis was analyzed by flow cytometry.

(d) MLKL-ND-HBD*-expressing NIH3T3 cells infected with lentiviruses carrying calreticulin shRNA (shCRT) or non-target shRNA (shSCR) were labeled with PKH26 and then induced to undergo necroptosis as described in **Fig. 1.** The necroptotic cells were co-cultured with CFSE-labeled NIH3T3 **(left panel)** or CD11c-marking BMDC **(right panel)**. Phagocytosis was measured by flow cytometry.

(e) CRT expression in indicated cells were analyzed by immunoblotting with the anti-CRT and anti-GAPDH antibodies.

(f) NIH3T3 **(left panel)** and BMDC **(right panel)** were incubated with BSA or CRT peptide (40μg/ml) for 30 mins before co-culture with necroptotic NIH3T3 cells treated as in **Fig. 1** for 4 hrs in the ratio of 1:1 (The final concentration of CRT peptide and BSA was 20μg/ml). Phagocytosis was analyzed by flow cytometry.

(g) Necroptotic or apoptotic NIH3T3 cells treated as in **Fig. 1** were pretreated with BSA or Annexin V (100μg/ml) for 30 mins before co-culture with NIH3T3 (WT) or Tim4-overexpressing NIH3T3 (Tim4) for 4 hrs in the ratio of 1:1 (The final concentration of Annexin V and BSA was 50μg/ml). Phagocytosis was analyzed by flow cytometry.

Data represent mean ± SD. ***$P < 0.001$; **$P < 0.01$; *$P < 0.05$; ns, not significant. Student's t test.

Supplementary Fig.S6

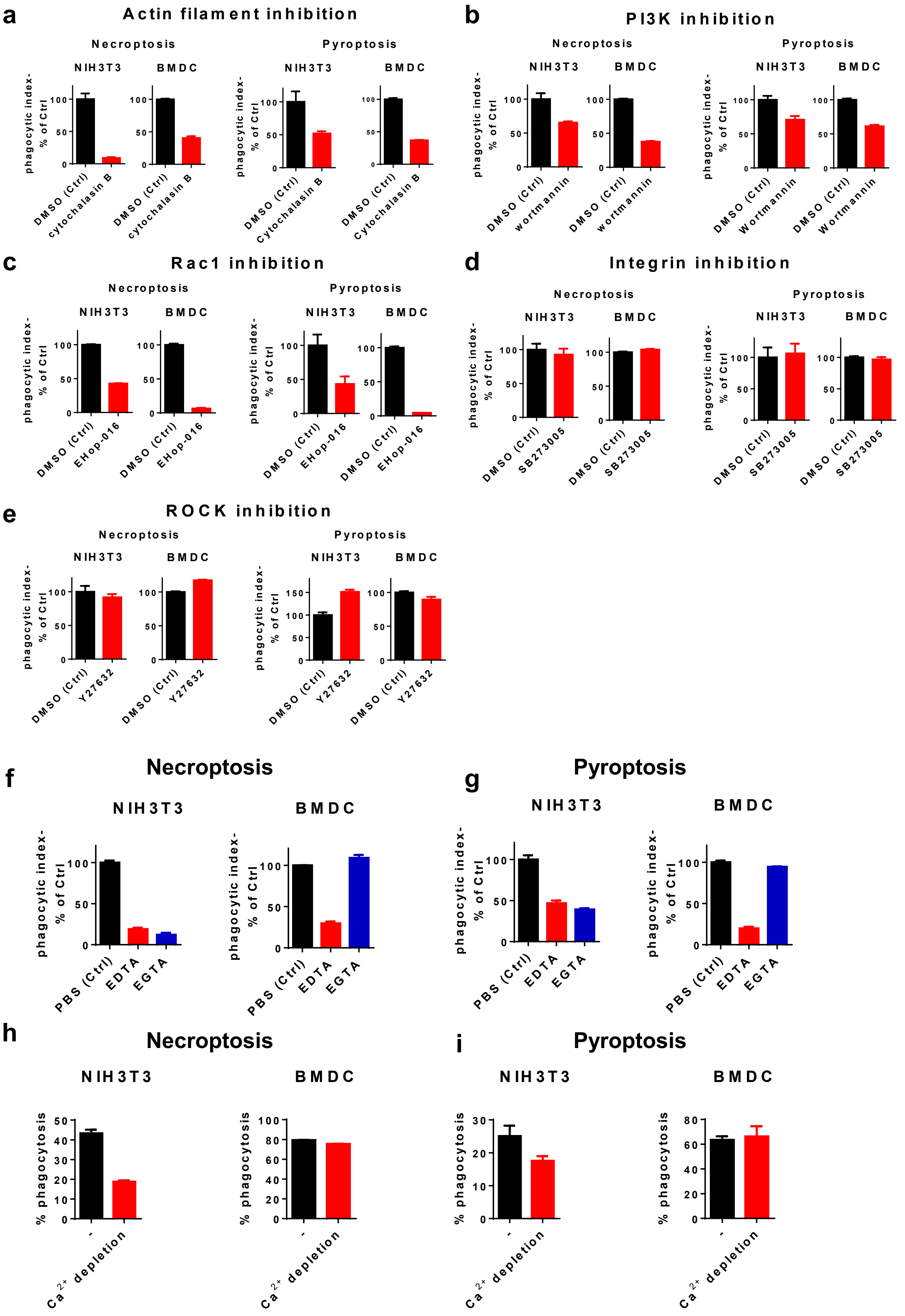

Supplementary Fig. S6 BMDC and NIH3T3 use different mechanism to engulf necroptotic cells.

(a-g) NIH3T3 or BMDC were pretreated with 40μM cytochalasin B **(a)**, 100nM wortmann **(b)**, 5μM EHop-106 **(c)**, 10nM SB 273005 **(d)**, 20μM Y27632 **(e)**, 2mM EDTA or 2mM EGTA **(f-g)** for 30 mins. And then they were co-cultured with necroptotic or pyroptotic NIH3T3 cells treated as in **Fig. 1** for 4 hrs. Phagocytosis was analyzed by flow cytometry. **(h-i)** NIH3T3 or BMDC were co-cultured with necroptotic or pyroptotic NIH3T3 cells in calcium normal or calcium depletion medium for 4 hrs. Phagocytosis was analyzed by flow cytometry.

Supplementary Movie S1

Engulfment of PKH26-labeled necroptotic L929 cells (red) by lifeact-EGFP-expressing MLKL KO L929 cells (green).

Supplementary Movie S2

Neighboring live L929 cells (green) ripped a necroptotic L929 cell (red) apart. Cells used here are the same as described in Supplementary Movie S1
